# Effect of Isothermal Conditions on the Charge Trapping/Detrapping Parameters in e-Beam Irradiated Thermally Aged XLPE Insulation in SEM

**DOI:** 10.3390/ma15051918

**Published:** 2022-03-04

**Authors:** Larbi Boukezzi, Sébastien Rondot, Omar Jbara, Sherif S. M. Ghoneim, Ahmed Boubakeur, Saad A. Mohamed Abdelwahab

**Affiliations:** 1 Materials Science and Informatics Laboratory, MSIL, University of Djelfa, Djelfa 17000, Algeria; larbiboukezzi@gmail.com; 2 Laboratoire Matériaux et Ingénierie Mécanique, UFR Sciences, Université de Reims Champagne Ardenne, BP1039, 51687 Reims, France; sebastien.rondot@univ-reims.fr (S.R.); omar.jbara@univ-reims.fr (O.J.); 3 Department of Electrical Engineering, College of Engineering, Taif University, P.O. Box 11099, Taif 21944, Saudi Arabia; 4 Laboratoire de Recherche en Electrotechnique (LRE), Ecole Nationale Polytechnique, BP 182 El-Harrach, Algiers 16200, Algeria; ahmed.boubakeur@g.enp.edu.dz; 5 Electrical Department, Faculty of Technology and Education, Suez University, Suez 43533, Egypt; saad.abdelwahab@suezuniv.edu.eg; 6 Department of Computers & Systems Engineering, High Institute of Electronic Engineering, Ministry of Higher Education, Bilbis-Sharqiya 44621, Egypt

**Keywords:** XLPE, SEM, thermal aging, trapped charge, secondary electron emission SEE

## Abstract

The effect of isothermal conditions on the trapping/detrapping process of charges in e-beam irradiated thermally aged XLPE insulation in scanning electron microscopy (SEM) has been investigated. Different isothermal conditions ranging from room temperature to 120 °C are applied on both unaged and aged XLPE samples (2 mm thick) by a suitable arrangement associated with SEM. For each applied test temperature, leakage, and influence currents have been measured simultaneously during and after e-beam irradiation. Experimental results show a big difference between the fresh and aged material regarding trapping and detrapping behavior. It has been pointed out that in the unaged material deep traps govern the process, whereas the shallow traps take part in the aged one. Almost all obtained results reveal that the trapped charge decreases and then increases as the temperature increases for the unaged sample. A deflection temperature corresponding to a minimum is observed at 50 °C. However, for the aged material, the maximum trapped charge decreases continuously with increasing temperature, and the material seems to trap fewer charges under e-beam irradiation at high temperature. Furthermore, thermal aging leads to the occurrence of detrapping process at high temperatures even under e-beam irradiation, which explains the decrease with time evolution of trapped charge during this period. The recorded leakage current increases with increasing temperature for both cases with pronounced values for aged material. The effect of temperature and thermal aging on electrostatic influence factor (*K*) and total secondary electron emission yield (*σ*) were also studied.

## 1. Introduction

Nowadays, renewable energy sources are a promising solution to preserve the environment. However, the big challenge that must be handled is that most of the hydro, solar, and wind sources are situated far from the urbanized areas, which means that the generated energy cost must be associated with the effective cost of the transmission system [1]. It is found that the use of a high voltage direct current (HVDC) system can reduce the energy losses to about 50% compared to the alternating current system (HVAC) [2]. For many cases, where the transmission system must be underground or crossing the sea (interconnection between countries for example), the use of the extruded cables with polymeric insulation presents a promising solution. At present, the dominantly used insulation is cross-linked polyethylene (XLPE). This material gained its place because of its superior dielectric and mechanical properties associated with its appropriate physicochemical characteristics [3,4]. In an HVDC application, the accumulation of space charge in the insulators is one of the serious issues that must be understood and controlled to ensure the safe operation of the HVDC system [5,6]. There is an interrelationship between the space charge accumulation and the premature aging of the insulators [7]. Although extensive research works have been conducted to evidence the existence of this interrelationship, it often remains poorly understood. One of the important conclusions is that the accumulation of charges in the cable insulation locally distorts the electric field, making the aging process faster [8,9]. Moreover, the aging of the material could generate physical and chemical defects which act as traps of the charge. The enhancement of generated traps with the aging process will further intensify charges accumulation [10].

It is well known that aging is an irreversible behavior of the cable insulation properties under long-term exposure to the operating conditions. The temperature is one of the most potent factors that could permanently present when the cable operates under normal service conditions (relatively low temperatures). However, under emergency conditions such as overloading or short-circuiting, the insulator is subjected to high temperatures. The presence of this high temperature can firstly undergo the so-called thermal aging, and secondly it has a direct effect on the trapping and detrapping dynamics of the space charge in the bulk of the insulator. Different studies have been conducted on thermal aging and temperature effect on polymers’ trapping and detrapping of charges. Chen et al. [11] have studied the effect of thermal aging on charge dynamics in the 320 kV HVDC XLPE cables. They have found that thermal aging facilitates the increase in trap energy density leading to the high ability of charge accumulation. In another research work, Liu et al. [12] have conducted thermal aging at temperatures above and below the melting peak of 160 kV XLPE insulation and studied the effect of the carried out aging on the distribution of space charges. The conclusion is that thermal aging improves the crystalline morphology of the XLPE at the early stage leading to the decrease in defects (traps) and space charge density. Nevertheless, at the end of the aging period, the total space charge would increase with the progressive degradation of the XLPE caused by long-term thermal aging. Ouyang et al. [13] have analyzed the effect of microstructure changes on the space charge distribution of XLPE during thermo-oxidative aging. They have deduced that shallow and deep traps have been introduced in the surface of XLPE by carbonyl groups and interfaces of crystal/amorphous, respectively. Thermo-oxidative aging at 160 °C dramatically decreases the deep trap density because of the destruction of spherulites, leading to the electrode injection enhancement. Li et al. [14] have examined the effect of 1-year laboratory-accelerated aging and 22 years in-service aging on the physicochemical origin of space charge dynamics in XLPE cable insulation. The significant finding drawn from their study is that the aging mode influences the trapping character. It is suggested that the deep traps are dominant in the aging process of 1-year laboratory-accelerated aged cable, according to slow accumulation of space charge. However, since space charge increases rapidly and then slowly for 22-year cable insulation in service; so, both shallow and deep traps are suggested to be effective. 

Moreover, the direct effect of temperature on the charge’s trapping and detrapping in XLPE insulation has been also investigated by many scholars in different ways. Many scholars have applied only isothermal conditions under DC stress to study the temperature’s effect on the charging process (trapped charges) [14,15], and others [16] have applied isothermal conditions under charging process to evaluate the trapped charge dependence on the applied temperature and non-isothermal conditions to evaluate the detrapping charges. In another work, presented in [17], only the non-isothermal treatment has been applied to stimulate the material to release the trapped charges under electron beam irradiation. Furthermore, the temperature gradient effect on the space charges distribution through the thickness of the insulation has been analyzed in [18]. The common conclusions from the above investigations are that the main temperature’s effect on the space charges dynamics is the enhancement of ionic dissociation of polar cross-linked by-products, charges injection enhancement from electrodes and enhancement of charges mobility. In addition, the amount of trapped charge decreases; however, the flowing charges increase with applied temperature which makes the detrapping process easier.

During the last few decades, many techniques have been developed and used to study the trapping and detrapping dynamics of space charges in polymers [19]. These techniques are based on the propagation of acoustic or thermal waves through the dimension of already charged sample. However, during the charging process, it is very difficult to record any information on the trapped charges evolution. For this reason, scanning electron microscopy (SEM) has attracted more attention of many researchers in recent years [20,21,22,23] to be investigated in the field of charges trapping and detrapping in polymers. The sample, under vacuum conditions in the SEM, is subjected to an electron beam irradiation of many kiloelectron volts. In this process, the sample is charged without electrode interfaces. Then, the electron beam is cut-off, and the released trapped charge can be evaluated. Thanks to a suitable device installed in the SEM, two currents can be measured together even during the charging or discharging process (dynamic measurement). The first current is called leakage current and represents the charges flowing through the sample’s surface to the grounded electrode. It can be used to evaluate the conduction process of the material. The second current is called displacement (influence or electrostatique induction) current and results from the trapped charges in the sample. 

The work carried out in this paper has two points of interest. The first is to study the effect of both thermal aging and isothermal conditions on trapping/detrapping processes in XLPE insulation under SEM environment. The adapted device in the SEM is associated with a heating system controlled by the PID regulator to obtain the specifically chosen testing temperatures. The consideration of both factors’ effects together on the trapping and detrapping of charges is poorly investigated in literature and our contribution can give more insights on this subject. The second point is the quantitative evaluation of both factor’s effects by estimation of trapped charge, leakage current, displacement current and secondary electron emission yield. 

## 2. Fundamental aspects

### 2.1. Electron Beam Irradiation of Dielectric Materials in SEM

The surface charging of dielectric materials can be performed by several methods based on different physical phenomena. Some of them are based on corona charging or DC stress charging. The other way to obtain charged dielectric materials is the bombardment with e-beam radiation in vacuum conditions of SEM. In this latter technique, a principal (primary) electron beam having few keV energy and few nA current intensity is injected into the material’s surface to deposit charges over a range of depths averaged in a few micrometers [24]. Therefore, negative and positive charges are built-up in competitive ways. The negative charges result from the incoming electrons’ trapping, and the positive charges result from the ejected secondary electron emission (SEE) that leave behind holes (positive charges) in the irradiated material. The non-trapped part of the emitted electrons and those generated by the SEE are drift through the sample to the ground generating the so-called leakage current (*I_L_*). This current contributes itself to trapped charge regulation at any time *Q_t_*(*t*).

The all out-coming currents must be balanced at all time and satisfy the following equation:

(1) $$I_{0}=\sigma I_{0}+dQ_{t}/dt+I_{L}$$

where *I*_0_ is the primary injected current by the beam, *σ = δ + η* is the total electron emission yield (*δ* and *η* are the secondary emission and backscattering electron coefficients, respectively), *I_L_* is the leakage current, *dQ_t_/dt* gives the trapping/detrapping rate during the charging/discharging process and *Q_t_*(*t*) is the trapped charge quantity at time *t* in the penetrating depth in the sample during the charging or discharging process.

### 2.2. Trapped Charge Measurement with SEM Device

**Figure 1:**
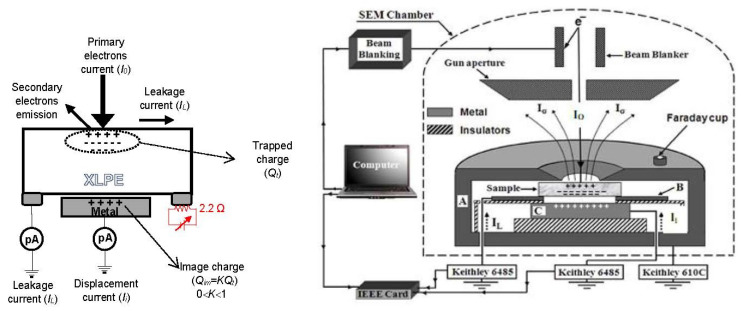
Sketching of the used adapted device in SEM. Reprinted with permission from Ref. [26]. Copyright 2022 Elsevier.

The evaluation of the trapped charge by the adapted device in the SEM is based on electrostatic influence phenomena (see sketch on left of Figure 1). If the irradiation is performed continuously with e-beam energy ranged between 5 and 30 keV, it was found that the negative trapped charge is the dominant one [25].

The buildup of trapped negative charge repels free electrons from nearby conductors in the SEM chamber, especially in the holder and the gun, and thus produces a positive countercharge (also called image charge (*Q_im_*) at the surface of these conductor parts (electrostatic induction or influence phenomenon). In most cases, the electrostatic influence is partial, so the trapped charge and the image charge are not equal, but they are related between them with a constant *K* (*K* ≤ 1) called the electrostatic influence factor as highlighted in Equation (2):

(2) $$Q_{im}=KQ_{t}$$

*K* is a calculable or measurable quantity [25] and is dependent on the sample geometry (thickness), sample properties (relative permittivity) and the electrical characteristics of the environment surrounding the sample. It was also found that this factor depends strongly on the applied temperature [27,28,29]. 

During the charging/discharging process, the variation of the trapped charge generates a new current called displacement (influence) current *I_i_*, independent on the leakage current. This current corresponds to the flowing current to the grounded electrode during the trapping of electrons in the sample. The following relationship translates what we have said above:

(3) $$I_{i}=\frac{dQ_{im}}{dt}=K\frac{dQ_{t}}{dt}$$

The good estimation of the trapped charge, at any time *t*, is conditioned by the determination of *K*. The experimental methodology adopted to determine the mean value of this factor is based on the simultaneous measurement of leakage and displacement currents during the discharging step. During this period, the e-beam irradiation is stopped (i.e., *I*_0_ = 0). Hence, Equation (1) becomes:

(4) $$\frac{dQ_{t}}{dt}=-I_{L}$$

Equation (4) assumes that any variation of trapped charge in the sample is neutralized only by the generated leakage current. The minus sign exhibits that both currents during the discharging period are opposite. 

If we replace Equation (4) in Equation (3), the value of *K* can be deduced and given by the following equation:

(5) $$K=-\frac{I_{i}}{I_{L}}$$

When the factor *K* is known, the time variation of the trapped charge can be calculated by the numerical integration of the displacement current as follows:

(6) $$Q_{t}\left(t\right)=\frac{1}{K}\int_{0}^{t}I_{i}\left(t\right)dt$$

In the end of this section, it is worthy to emphasize two main points. The first is the difference between the used e-beam irradiation method in SEM to inject charges into dielectrics and the other common methods. The injection of charge in this method escapes any contact with the sample (no additional effect of interfaces), whereas the injection of charges is performed under electric field between two electrodes in full contact with the sample in the other methods. Moreover, the electron beam can be positioned to the chosen position with great precision (local irradiation) and several penetration depths of electrons in the irradiated sample can be achieved by changing their primary energy. The second point is that during a few decades of time, the developed theoretical aspect has been validated in a consistent experimental test with the buildup arrangement in a SEM on a large variety of materials ranging from glasses [22,27] passing through polymers [20,23,25] and ending with nano-composites [21,26].

## 3. Materials and Methods

### 3.1. Materials and Samples Preparation

The chosen material in this study is the cross-linked polyethylene 4201 Extra Clean supplied by Union Carbide Corporation (Houston, TX, USA). This material is common in the manufacture of 18/30 kV high voltage cables. In its granulate form, the main material is filled with 2% of dicumyl peroxide (DCP) as cross-linking reactive and 0.2% of Irganox 1035 as antioxidant. The preparation of test samples is done with a heat press apparatus under 180 °C and 300 bars. These conditions are applied for 10 min to achieve the fully cross-linking reactions of the samples. The obtained samples have 2 mm thickness.

### 3.2. Methods

#### 3.2.1. Thermal Aging Test

The obtained samples of XLPE insulation have been aged in an air forced circulating oven. The selected aging temperature is maintained by self-regulating process of the oven within a precision of ±2 °C. The aging temperature is 140 °C and the aging duration is 1500 h. The motivation to choose this aging temperature is argued by its situation in the emergency operating temperatures for XLPE material when used as insulation in power cables. The aging period is chosen to be 1500 h because at this aging time the degradation of the material is apparent and further aging time is not needed. 

#### 3.2.2. Adapted Device in SEM and Measurements

The adapted device in SEM (T330 type fabricated by JEOL Ltd., Tokyo, Japan) measuring simultaneously and separately both currents (leakage current and displacement current) is composed of three main parts described extensively in previous papers [23,25] and sketched in Figure 1.

Here, we briefly recall these parts and highlight the modification introduced into the device to study the effect of isothermal conditions (constant temperature) on the trapping and detrapping process of the XLPE insulation. The first part of the used device is a cylindrical grounded metallic enclosure that acts as a shield to avoid the emitted electrons from the SEM chamber walls to be added into the measured currents. This enclosure has a circular hole of 6 mm in diameter at its upper face. A small Faraday cage was stuck on this enclosure and used to measure the primary current *I*_0_. The second part consists of a copper disc that acts as an image charge probe situated above the sample. This disc is not in contact with the sample and is plugged to the ground via a picoammeter (Keithley 6485 fabricated by Solon, OH, USA) to measure the influence current *I_i_*. The third part is a brass electrode shaped as a disc containing circular opening of 10 mm in diameter. This electrode is in good contact with the sample and measures the leakage current *I_L_* via another picoammeter (Keithley 6485). The used picoammeters are able to detect currents up to 10^−15^ A with an integration time of 0.2 s. The good contact between the sample and this electrode is ensured with a silver paint stick.

Thanks to the heating system associated with the standard adapted arrangement, the effect of the isothermal conditions on fresh and aged samples of XLPE can be studied. The heating system comprises a ceramic resistor of 2.2 Ω producing high temperatures under specific applied voltage and current. This resistor is stuck on the electrode in contact with the sample (see Figure 1). In addition, a PID regulator is associated with the heating system to obtain stabilized testing temperature in the SEM chamber. 

The experimental procedure followed in this study is as follows: first, samples with 14 mm in diameter are cleaned in alcohol bath inside an ultrasonic tank during 15 min. After that, each selected sample was introduced in the measurement device in SEM chamber and heated to the desired temperature. The selected temperatures are: room temperature, 50 °C, 80 °C, 90 °C and 120 °C. Each temperature is maintained constant during charging and discharging period. This wide range of applied temperatures covers the semi-crystalline state (temperatures below the melting pick of XLPE) and the polymer’s molten state (temperatures above the melting temperature). Sample is kept under a 10^−6^ Torr and selected temperature for 30 min to achieve heat spreading equilibrium in all sample volume. Once the equilibrium is reached, the sample is locally irradiated with e-beam spot (size 0.5 μm) having 15 keV as primary energy and 2.5 nA as primary current. After a few hundred seconds under the charging process, the e-beam is cut off at time *t_off_* and the discharging process starts. During both processes, the time variation of leakage and displacement currents is recorded simultaneously and separately by the associated picoammeters. 

## 4. Experimental Results and Discussions

### 4.1. Effect of Isothermal Conditions on the Currents

#### 4.1.1. Fresh Samples

**Figure 2:**
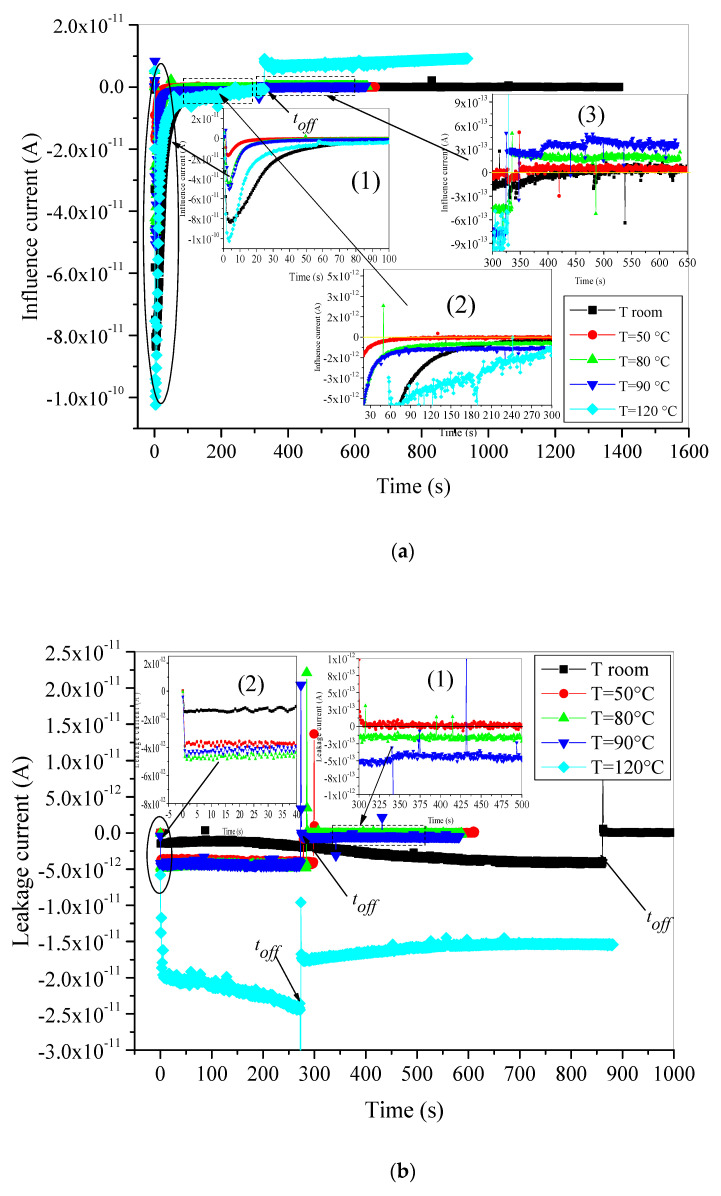
Time evolution of: (**a**) displacement current, (**b**) leakage current, during and after e-beam irradiation of unaged XLPE material.

Fresh samples of XLPE insulation are irradiated with 15 keV e-beam primary energy and 2.5 nA e-beam primary current. The time evolution of both leakage and influence currents are recorded with the picoammeters during and after irradiation in SEM for each applied temperature. Figure 2a,b display the recorded currents. Before analyzing the presented results in these figures, we should bear in mind that during the irradiation period the injected electrons with e-beam are subjected to two sources of energy, electrical energy (e-beam energy) and thermal energy (temperature). Hence, the behavior of the trapping process may be influenced by the co-existence of both energy sources. By examining the curves of Figure 2a,b, we can see that during irradiation, leakage and influence currents are negative but decay completely in different ways. For each isothermal condition, the influence current decays in fast way (seem to be exponential) to reach very small negative values after very short times (generally few seconds) (see inset 1 in Figure 2a). The reached values remain constant except in the case of test temperature 120 °C. For this temperature, the courant *I_i_* seems to continue its decrease (in absolute value) (see inset 2 in Figure 2a) until the discharging process starting at *t_off_*. The reached low value can be practically equal to zero, like in the case of the charging process at room temperature and at 50 °C, or different to zero when the samples are irradiated at relatively elevated temperatures (see inset 2 in Figure 2a). At the beginning of irradiation, the influence current gets its maximum value, which means that the charges trapping rate is the highest. Afterwards, the trapping rate decreases owing to the progressively filling of traps (preexisting traps and traps created by irradiation) by injected electrons on one hand and on the other hand to the secondary electron emission increase induced by the negative trapped charge [30]. At room temperature and 50 °C, the dominant source of energy is the e-beam energy, and the thermal energy can be neglected, so when all the convenient traps are completely filled, the trapping of charge is stopped and the trapping rate $dQ_{t}/dt=0$. Thus, the corresponding measured influence current also is null.

At temperatures 80 °C and 90 °C, where XLPE always maintains its semi-crystalline structure, both injected and existent electrons may gain more sufficient energy from the applied temperatures. This supplementary energy may have two effects: First, it makes the trapping process faster as can be seen in the inset 1 of Figure 2a. Second, it helps many trapped or already existing electrons to break free by going beyond the depth of the traps. In consequence, their located traps become free and can be filled by other injected electrons. The number of detrapped electrons increases with increasing temperature. 

We may admit that in this range of temperatures, the trapping rate exceeds the detrapping rate. The difference between both rates can be assumed as constant, which explains the constant values of the low negative currents when the steady-state is reached. In addition, the influence current behavior at 120 °C is quite different because of the changes in the XLPE structure at this temperature. It is well known that the XLPE becomes completely molten at high temperatures above the melting peak of practically 105 °C (determined with deferential scanning calorimetric (DSC) in our previous former publication [31]). The amorphous character of the material at 120 °C leads to the changes in the traps density, traps depth and nature. The influence current at 120 °C decreases continuously, and no steady state has been reached. It seems that the change in sign of *I_i_* in this period is highly probable. The behavior of this current at this temperature can be assigned to two causes.

The first one is the energy of the injected electrons (by the e-beam) that increases under high temperature (thermal energy). Hence, they can escape to the trapping process or have a high probability of detrapping if trapped in the polymer.

The second reason is the changes in the traps’ density and depth of XLPE under high temperatures above the melting peak. It is worth noting that the presence of two phases (amorphous and crystalline) in the insulating material’s structure, such as XLPE, presents an aggregation of deep and shallow traps [32]. The presence of chemical defects and additives in the amorphous regions (enlarged by heating at very high temperatures) contributes to the enhancement of shallow traps [33], making the detrapping process of energetic electrons very easy. These two main reasons may make the detrapping process faster than the trapping process and give the decreasing trends of the influence current. This phenomenon will be discussed deeply in the next section when we evoke the effect of temperature on the dynamic trapping of charges. 

At *t_off_*, the e-beam irradiation becomes *off*, and the discharging process starts. When e-beam is stopped, no new electrons are injected in the material. Only thermal energy caused by the applied isothermal conditions is exercised on the already trapped electrons in this step. Therefore, we expect that detrapping process is the dominant one. At room temperature and at 50 °C, no detrapped charges occurred because of the relatively low applied temperature, so the resulting influence current maintains its null values. It has been evidenced in previous papers that XLPE has a strong ability to store charges at room temperature after stopping e-beam irradiation [17,23]. When the applied temperatures rise (80 °C and 90 °C), we notice an abrupt change of sign in the influence current that becomes positive. Nevertheless, no bulk discharge is observed. The bulk discharge brings main part of trapped electrons to leave material surface in very short time leading to a fast decrease in the detrapping rate. In most studied materials [26,27,28,29], the detrapping rate decreases exponentially owing the influence current to take the same trends. Consequently, the influence current preserves moderate constant values for both temperatures, which means that detrapping process under high temperatures (below the melting peak) is constant. Only few electrons with enough energy can the overcome traps’ depth. Therefore, the detrapped electrons reach the sample’s surface and are evacuated to the ground as part of leakage current. This point will be confirmed later when we speak about leakage current. The discharge process at 120 °C is intensive and is different from the other temperatures. The displacement current decreases from a maximum positive value at the beginning of this step to reach a minimum after 50 s and then increases progressively. 

When it comes to the leakage current, it has been observed that during the irradiation period, leakage current increases continuously (in absolute value) to finally reach a saturation value (*I_LS_*) (see the inset one in Figure 2b). This behavior is observed only for test temperatures ranging from room temperature to 90 °C. It is noticeable that the higher the temperature the greater *I_LS_* values. The obtained results of *I_L_* are in concordance with those of *I_i_*. This concordance is also observed for test temperature 120 °C where the increasing of detrapped electrons leads in one hand to the continuous decrease in displacement current and expected changes of its sign as we have already stated. On the other hand, they contribute to the enhanced values and the increasing trend of the leakage current (in absolute values). Furthermore, this behavior can be related to the surface conductivity activation by the temperature, which leads to more important evacuation of free electrons created within the sample surface by the detrapping process to the ground [29]. 

Once the e-beam is blanked, leakage current maintains its negative sign and fall to lesser values according to each applied isothermal condition. At room temperature and at 50 °C, the discharge process is quasi-nonexistent and leakage current falls immediately to zero. It indicates the high charge stability in the XLPE under low temperatures. The charges are captured in permanent trapping states, limiting the possibility of observation of the discharging phase (see inset 2 in Figure 2b). If we assume the release of a meager amount of electrons from dominant existing deep traps, these electrons will be trapped by other trapping states, and the motion of charges is completely stopped. The phenomenon may be interpreted with the developed multiple trapping model [34]. The pursuit of that motion of an electron located in a localized state is only possible when the electron experiences a jump to the extended states by thermal activation energy. The necessary thermal activation energy is available when the applied testing temperature is relatively high, as we can see in the case of 80 °C and 90 °C where few electrons acquired enough thermal energy to reach, after a series of jumps, the surface of the material. The evacuation of these electrons is the origin of the observed small negative leakage current as we can see in inset two of Figure 2b. When the applied temperature exceeds the melting peak (120 °C), the leakage current falls immediately to a new negative value because of the absence of the intrinsic electrons coming from the e-beam irradiation. The new evolution of this current is due only to the progressive evacuation of the trapped electrons caused by the detrapping process under relatively high thermal activation energy. We guess that both leakage and influence currents behave similarly according to the given Equation (5) in Section 2.2. If we assume that the influence factor *K* keeps its value constant, the increase in the former leads to the increase in the other (in absolute value). However, as we observe in Figure 2a,b, the influence current increases faster than leakage current, which means that only one part of the detrapped electrons is evacuated out of the sample. The other part is retrapped again by other relatively deeper traps. 

#### 4.1.2. Aged Samples at 140 °C

**Figure 3:**
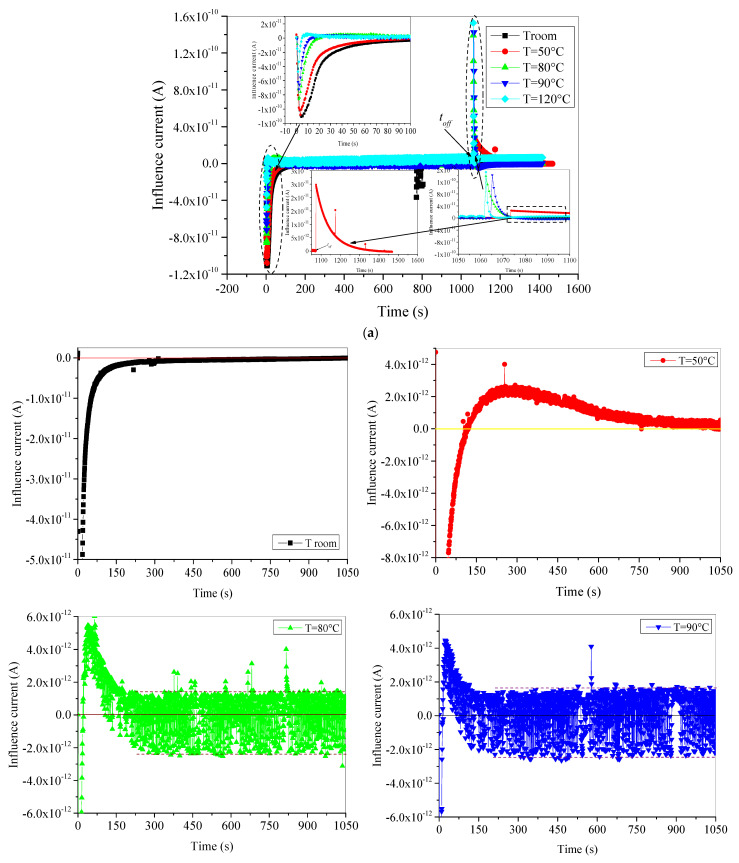

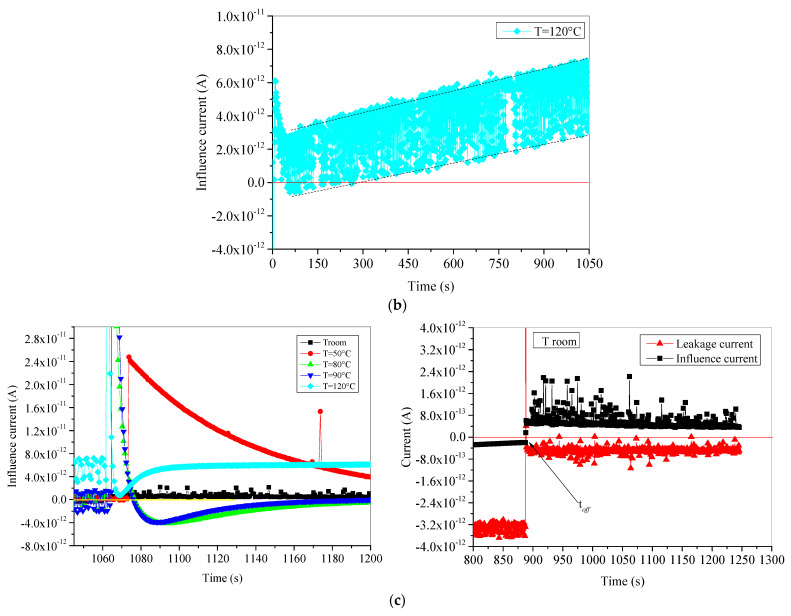
Time evolution of displacement current of aged XLPE material: (**a**) both charging and discharging periods together; (**b**) expended form of charging process for each temperature alone; (**c**) discharging process after e-beam irradiation is cut-off.

**Figure 4:**
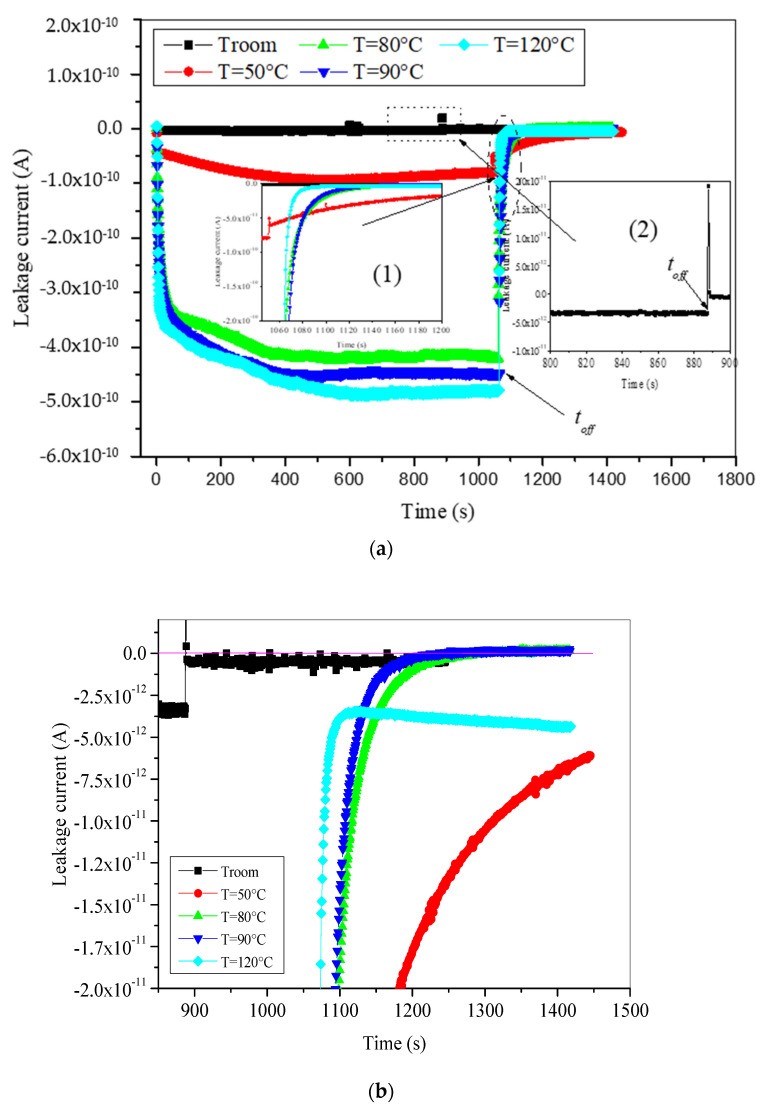
Time evolution of leakage current of aged XLPE material, (**a**) both charging and discharging periods, and (**b**) enlargement of discharging period.

The conducted experiences for aged samples are similar to those for unaged samples (irradiation under 15 keV e-beam primary energy and 2.5 nA primary current). The obtained results are depicted in Figure 3 and Figure 4. 

Before starting our analysis, we should state here that thermal aging at 140 °C can be considered as very harsh stress. This stress can cause serious alteration either on the macroscopic and microscopic characteristics of XLPE insulation. Regarding the trapping and detrapping process, thermal aging at high temperature affects the distribution and nature of traps. If we believe in the earlier finding of many scholars ascertaining the co-existence of deep and shallow traps in the XLPE structure [33,35,36], 1500 h of aging at 140 °C brings on one hand more shallow traps than deep traps, as highlighted in many types of research [11,12,13], and on the other hand an increase in the conductive character of the material [37], leading to faster evacuation process of charges. Furthermore, this new structure greatly affects the behavior of both influence and leakage currents under different applied isothermal conditions, as we can see in Figure 3 and Figure 4. 

In Figure 3a is depicted the influence current when e-beam irradiation is *on* and when it is *off*. The given insets with this figure concern this current’s behavior at the beginning of the charging process (inset 1) and discharging process (inset 2). It is very clear from these insets that the influence current is negative at the beginning of irradiation and positive when the irradiation is stopped. In both cases, this current decays fast from a maximum to zero. Moreover, the reached maximum and the decaying speed are very influenced by the applied test temperature. To show more details on this current behavior, we have presented in Figure 3b,c an exploded form of the previous figure. In Figure 3b, we have presented the charging process giving the evolution of influence current during all the periods where the e-beam irradiation is *on* for each test temperature alone. The discharging process is given in Figure 3c. From this exploded form, it is clear that at room temperature, the influence current takes the usual form [17]. It starts its evolution from a maximum (in absolute value) negative value and decays rapidly to reach zero after a few hundred seconds. This behavior is the common trapping process observed in the SEM when insulating materials are subjected to the e-beam irradiation. However, when the applied temperature is rising, we have observed two interesting phenomena:

First, the influence current starts decreasing from the maximum value (in absolute value) with a negative sign and then becomes positive after a relatively short time. This time is shorter as the applied test temperature is higher. As becoming positive, the influence current increases to reach a maximum and then decreases. The negative part of the curve concerns the trapping process of the injected electrons by e-beam irradiation. The higher the applied test temperature is, the faster the trapping rate is. Under specific applied isothermal conditions, part of the trapped charge is detrapped despite the permanent existence of the e-beam irradiation, leading to a positive evolution of the current. We should note here that time constant of the detrapping rate is larger than time constant of the trapping rate. Nevertheless, the maximum trapping rate is greater than the maximum detrapping rate. The appearance of detrapping process at temperatures above 50 °C in this step proves that a part of trapped electrons is trapped in very shallow traps generated in the surface of samples by the aging process. 

The second point concerns the presence of oscillations in the recorded influence current. These oscillations are little in the case of test temperature 50 °C and become very intense for other high temperatures and limited with an overlap of 4 × 10^−12^ A. The presence of oscillations indicates that the trapped charge in shallow traps is not stable when submitted to electrical and thermal energies. Both energies exert two opposite forces on the trapped charge, leading to successive processes, trap–detrap–retrap, hence the generation of oscillations in the current shape.

Furthermore, after detrapping of all corresponding trapped charge, the influence current decreases toward a steady state around zero. The steady state is reached after 1050 s, 200 s, and 150 s for test temperature 50 °C, 80 °C, and 90 °C, respectively. In the case of 120 °C, the evolution of influence current reaches a positive minimum after 50 s and then continuously increases.

To analyze the discharging process, we have presented an enlarged show of influence current during the discharging process in Figure 3c. When e-beam irradiation is switched off, the discharge process occurred for all temperatures, even at room temperature, without any oscillations at high temperatures, like in the charging process, whereas at room temperature, a small amount of trapped charges is detrapped, generating a very low positive influence current (maximum does not exceed 4 × 10^−13^ A) which decays very slowly, as shown in Figure 3c. When the applied test temperature increases, the amount of detrapped charges becomes more and more bulky and the detrapping rate is more and more fast. Thus, influence current suddenly decays positively in exponential form with more important magnitude as temperature is higher (see the insets 2 and 3 in Figure 3a). From the enlarged show depicted in Figure 3c, we can observe that in the case of applied test temperatures 80 °C and 90 °C we have a reverse behavior than the once observed when the irradiation step is started. Here, influence current decreases positively (detrapping process of electrons) at the beginning of discharging step and then starts to take negative values. By changing the sign, the current continues its increasing (in absolute value) and then decreases to reach a steady state around zero value. This is a typical behavior of charge trapping process. This latter behavior may be due to the retrapping of small part of the bulky detrapped electrons in existing deep traps in the surface of the material searching to achieve some equilibrium of the charge reparation in the sample. This rearrangement of charges in the bulk of the material is local and does not have any contribution on the conduction process because the evolution of leakage current does not react with this tilting from detrapping to retrapping process of electrons. Conversely, the behavior of the influence current in the case of applied temperature 120 °C has an effect on the behavior of the leakage current. For this temperature, after a fast exponential decrease in the influence current to reach a positive minimum, it starts to increase again permanently. This permanent increase is commonly due to the detrapping of electrons under high thermal activation energy. The detrapped electrons have sufficient energy to reach the surface of the sample and be evacuated to the ground via the picoammeter recording the leakage current. Hence, leakage current will have also an increasing trend as we will see in the next paragraph. 

Figure 4 presents leakage current during both charging and discharging processes. This current evolution during the charging process is quite similar to that observed in fresh samples with very pronounced high values, especially at high temperatures. The enhanced values of leakage current after aging are caused probably by many factors as follows: First, the augmentation of the material conductivity caused by the thermo-oxidation degradation. It was found previously that the volume resistivity of XLPE insulation decreases from ~10^14^ Ω·cm to ~10^12^ Ω·cm [37,38]. Second, the reduction in the crystallinity [31,39] and the cross-linking degree [40] facilitates the creation of a lot of easy pathways in the low density regions of the material, hence enhancing the motion of free electrons. Third the enhancement of detrapping process caused by the high thermal activation energy of electrons which are trapped in intensely existing shallow traps created by the aging process in the surface of the material.

It is worthy to note here that leakage current during the irradiation period does not present the noisy behavior as the influence current. We have said that the noisy behavior in the influence current concerns the self-rearrangement of trapped charges by the effect of both e-beam energy and thermal energy. 

Furthermore, when the e-beam irradiation is blanked the leakage current drops immediately (in absolute value) from 3.5 × 10^−12^ A to 4 × 10^−13^ A at room temperature, and then decreases very slowly as we can see in Figure 3c. Meanwhile, the leakage current decays negatively at high temperatures in exponential form (see the enlargement view of the discharging period in Figure 4b). As the temperature is higher, the decaying rate is faster. At temperatures ranging from 50 °C to 90 °C, the leakage current evolution reaches a steady state around zero value. At 120 °C, this current decreases to a minimum close to 3.5 × 10^−12^ A (in absolute value) after few tens of seconds, and then starts to increase again in correlation with the influence current increase (Figure 3c). 

### 4.2. Effect of Isothermal Conditions on the Trapped Charge Dynamics

#### 4.2.1. Fresh Samples

**Figure 5:**
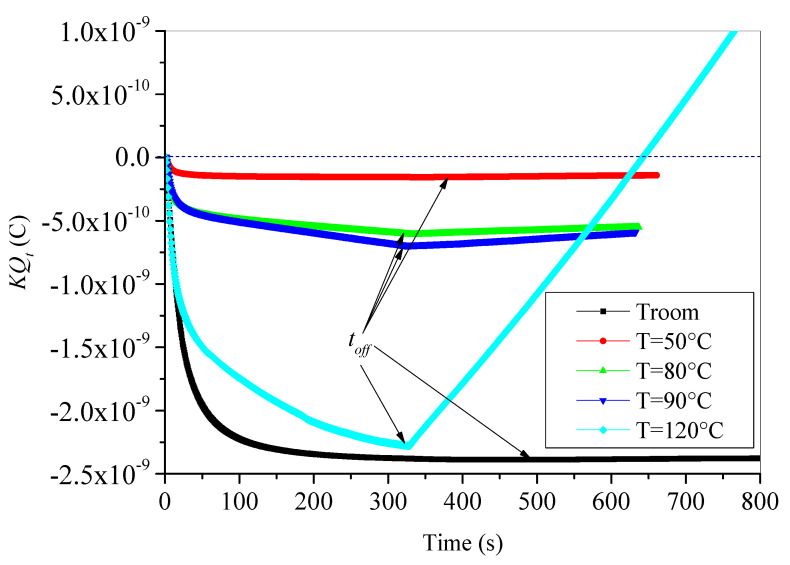
Time evolution of trapped charges in unaged XLPE material during and after e-beam irradiation.

The trapped charge dynamics under isothermal conditions in XLPE are governed by the trapping and detrapping processes. It is well known that the trapping process of electrons leads to negative charge formation and the detrapping of electrons from the traps leads to positive charge (holes) formation. Hence, the total charge sign results from the taken part of each process. In SEM, the evolution of trapped charge dynamics is evaluated both during e-beam irradiation (charging step) and after switch-off of e-beam irradiation (discharging process). By using the time-resolved characteristic of influence current, the trapped charge can be deduced from Equation (6) with simple numerical integration. For unaged (fresh) samples, the time dynamic evolution of trapped charge during and after e-irradiation, at different temperatures, is depicted in Figure 5. With the onset of e-beam irradiation, a negative trapped charge is a buildup. The buildup charge increases with time at the beginning of electrons injection in exponential shape for all test temperatures and behaves differently after that. At room temperature and at 50 °C, the negative buildup charge reaches a saturation value (steady-state); however, at 80 °C and 90 °C, it continues its increase linearly. For 120 °C, the increase in trapped charge takes always the exponential form. 

Let us now analyze the behavior of trapped charge when the discharging process is started. At *t_off_*, e-beam irradiation is stopped, the trapped charge behaves differently to the charging process. At room temperature and 50 °C, no discharging process has occurred, and the reached values at the steady state are maintained during the whole discharging process. However, the trapped charge decreases linearly (in absolute values), keeping its negative sign for test temperatures 80 °C and 90 °C. In the case of 120 °C, the linear decrease in trapped charge is faster and becomes positive after practically 300 s after *t_off_*. The change of sign of trapped charge means that the detrapping process of electrons is dominant, and many holes (positive charges) are created in the surface and the bulk of the sample under the effect of high temperature. 

#### 4.2.2. Aged Samples at 140 °C 

**Figure 6:**
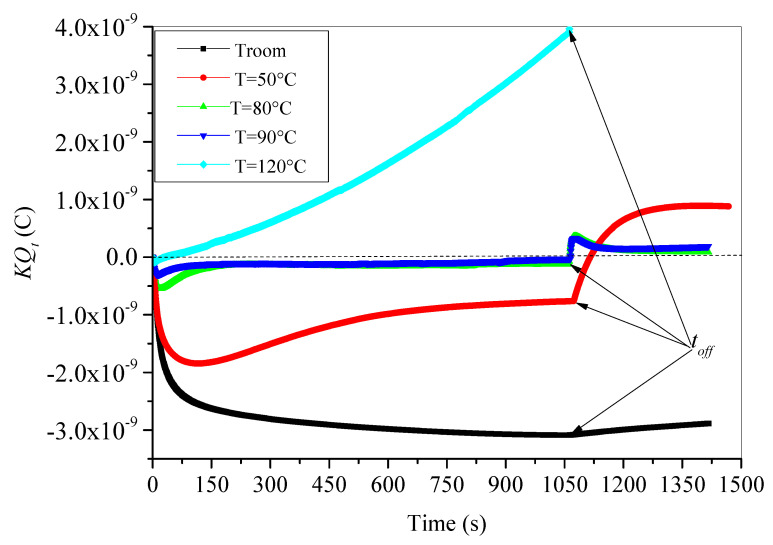
Time evolution of trapped charges in aged XLPE material during and after e-beam irradiation.

It is worthy to recall here that behavior of trapped charge for aged material is influenced by both temperature and thermal aging. Therefore, temperature enhances detrapping process aptitude of electrons from traps; meanwhile, thermal aging brings additional physical and chemical defects. These defects act as shallow and deep traps. It was highlighted by many researchers that traps generated by aging at relatively high temperature are shallow traps [11,12,13]. Keep this in mind when discussing the presented results in Figure 6 regarding the time evolution of trapped charge in XLPE samples aged at 140 °C, either during irradiation step or after irradiation. 

During irradiation step, an increase (in absolute value) of negative trapped charge is observed at the onset of electron irradiation subsequently. The increase in trapped charge reaches a maximum after a critical time *t_c_* and then trapped charge decreases again. This trapped charge versus time behavior was observed for all test temperatures except at room temperature where the trapped charge does not decrease but continuously increases linearly.

**Figure 7:**
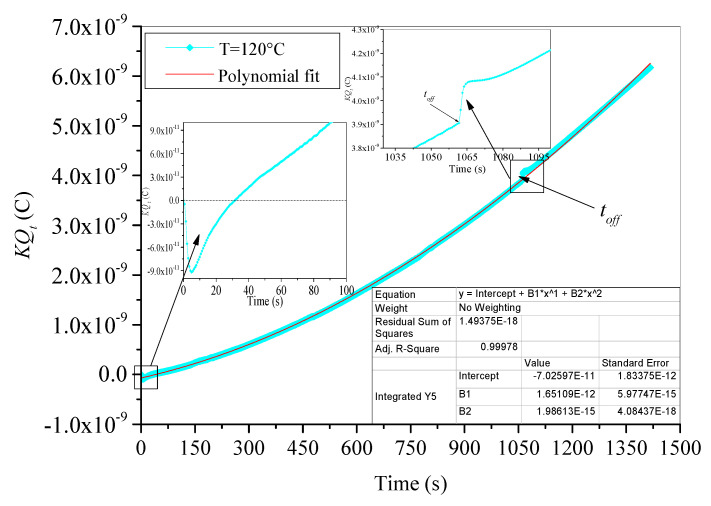
Fitting of trapped charge in aged XLPE material at 120 °C.

The critical time corresponding to a maximum trapped charge decreases as test temperature increases. For temperatures ranging from 50 °C to 90 °C, after a decreasing trend beyond time *t_c_*, the trapped charge preserves its negative sign and tries to reach a negative steady state (saturation). The steady state is reached faster as the test temperature is higher. In addition, the value of trapped charge at steady state is higher at 50 °C than at 80 °C and 90 °C. This means that the amount of detrapped charges caused by the thermal activation at 80 °C and 90 °C is greater than that caused by thermal activation at 50 °C. It is worthy to note here that the remaining trapped charge at the steady state is trapped in relatively deeper traps. Moreover, the trapped charge at 120 °C decreases continuously after *t_c_* (here *t_c_* equals only 5 s), becomes positive after 30 s, and then continues its positive increase in polynomial trends as we can see in Figure 7.

Moreover, trapped charge behavior during irradiation step under applied test temperatures is monitored by the concurrent effects of both trapping and detrapping processes. The increase in the negative trapped charge (in absolute value) means that the trapping process is the dominant one. This behavior is observed at the beginning of irradiation step for all test temperatures. Indeed, its supremacy is observed when the test is carried out at room temperature. At room temperature, the thermal energy supplied to the trapped electrons is not enough to achieve a massif detrapping. On the other hand, if trapped charge is negative and decreases (in absolute value), the detrapping process governs the process. This behavior is observed after the critical time *t_c_* for all elevate temperatures. The delay of detrapping process governance than trapping process is evident because the time constant to activate electrons with electrical energy (supplied by e-beam irradiation) is lower than the time constant of thermal activation. The alternating dominance of both processes is a way to search some equilibrium of charge repartition in the bulk of XLPE samples. When the equilibrium is reached, a steady state is observed and both trapping and detrapping processes have a same rate. This behavior is observed for test temperatures 50 °C, 80 °C and 90 °C. Furthermore, if the trapped charge is positive and increases, as in the case of test temperature 120 °C, this suggests that detrapping process is highly prevailing. At 120 °C, the material is completely molten, and the supplied thermal energy is enough to cause the following: on one hand, a fast detrapping process of trapped electrons; on the other hand, breaks of the holding bonds allowing electrons to be free and evacuated easily leading to extensive positive charges (holes) formation [15].

After irradiation, trapped charge is submitted to a pure detrapping process caused by thermal energy under a specific applied isothermal condition. Three behaviors can be observed in Figure 6. The first behavior concerns room temperature where trapped charge decreases (in absolute value) in linear trends, keeping its negative value. The slight linear decrease is assigned to the occurrence of detrapping process at room temperature with a very slow rate (only few electrons can be detrapped under room temperature). The second behavior concerns temperatures ranged from 50 °C to 90 °C where trapped charge subsequently changes its sign from negative to positive. The sign’s change is faster as the temperature is higher. As trapped charge becomes positive, it increases to a maximum and decreases after that with inverse exponential behavior to reach a positive steady state. The steady state is reached faster as the temperature is higher. The highly suggested explanation here is that when e-beam irradiation is stopped, only temperature affects trapped electrons; hence, the almost trapped electrons become energetic and leave their traps, at the same time leaving behind holes, i.e., positive charges. This process of positive charge generation explains the increasing trend at the beginning of discharging process just after time *t_off_*. Moreover, part of the detrapped electrons did not reach the outside of the sample, but they have been retrapped again by relatively deeper traps in the surface of the sample. The statement that deep traps are located at the surface and traps become shallower with increasing depth in the material was evidenced by Seggern [41]. While retrapping this part of electrons, a retention charge contributes to electron/hole pairs recombination, reducing the generated positive charge. The consequence is the observed decrease in the generated positive charges before steady state. When retrapping process is stopped and all escaped free electrons left the surface of sample, a steady state is reached. Additionally, the third behavior concerns the evolution of trapped charge at 120 °C, where the shutdown of e-beam irradiation generates only a small stepwise change in the time evolution of charge, as we can see in the inset in Figure 7. The generated charge always follows its polynomial increase. 

## 5. Discussion

In-depth discussion of the above results needs additional analysis on the effect of applied isothermal conditions and thermal aging on some complementary parameters characterizing trapping and detrapping processes of charges in XLPE insulation. Such parameters concern the maximum trapping rate (*I_imax_*), the leakage current at steady state (*I_LS_*), the influence factor (*K*), the critical time (*t_c_*), the maximum trapped charge during irradiation process (*Q_tmax_*) and the total electron emission yield (*σ*).

Before evoking all these issues, we start with a very important statement concerning the morphology of XLPE insulation regarding its relationship with temperature and thermal aging. When cooled from its melting state, XLPE takes its definitive cross-linked semi-crystalline structure, where ordered crystalline lamellars (spherulites) are inter-phased by amorphous regions; hence, a lot of interfaces crystal/amorphous are created. The oxidation stability of XLPE insulation is guaranteed by incorporating antioxidants in the materials during its manufacturing process. Thereby, this complex structure presents structural defects acting as traps of charge and significantly influences the trapped charge distribution. These traps have a wide range of distribution regarding their density and their energy. It has been evidenced in many studies that existing traps in XLPE materials are categorized into deep traps and shallow traps [13,35]. With no further thermal treatment (degasing process or thermal aging), the density of deep traps is greater than shallow traps density [35] which means that almost of the mentioned imperfections and additives likely behave as deep traps. Mainly, the existing deep traps are located in the crystalline parts, while in the amorphous regions there are more shallow traps because of the disorder caused by the existence of many shorter chains, chain branches and cross-linking sites. The cross-linking is an important process in XLPE cables manufacturing and further may affect traps distribution in the material. On one hand, with the onset of the cross-linking process, the molecular chains in the amorphous region of the material are gradually cross-linked to form a network structure which will enhance barriers for electron migration. Consequently, the higher the cross-linking degree is, the greater the number of deep traps, as ascertained by Yan et al. [35]. On the other hand, the peroxide cross-linking process leads to the formation of the so-called cross-linking by-products such as α-methylstyrene, acetophenone, and cumyl alcohol, which are volatile species and create traps with energy around 1.07 eV as traps depth [35]. Moreover, Meunier et al. [32] have found that the interfaces crystal/amorphous are the highly probable locations to provide deep traps having energy levels around 1 eV. They have also ascertained that chemical defects like polar groups, such as conjugated double bonds, hydroxyl, and carbonyl functions exist around the chains in the crystalline region. These chemical defects in crystalline and amorphous regions can contribute to deep traps of 1 eV in polymeric materials [42]. Therefore, this complex aggregation structure of XLPE after immediate cables manufacturing favors the dominance of deep traps over shallow traps. In addition, when an incident electron is trapped in one trap, its time residence depends on the trap’s nature. It is well known that carriers trapped in deep traps have larger time residence than those trapped in shallow traps, and their detrapping needs enough energy to escape from the trap. Therefore, behavior of trapped charge is extremely related in one part to the nature and distribution of traps in XLPE structure and in the other part to the trapping or detrapping process. It is well known that the detrapping process may be attributed mainly to the thermal detrapping [33] when the applied electric field is not enough to activate ionization and tunneling. It is the case with XLPE cables where the flowing of current through the conductor leads to insulation temperature increase according to Joule’s law. The effect of temperature on charges accumulation and transport in XLPE insulation has been investigated by many researchers [15,43]. Their main conclusions supporting our earlier finding presented in Section 4 are the following:

- The temperature has numerous effects on space charge dynamics, such as enhancing ionic dissociation of polar cross-linking by-products and increasing of electrons energy, enabling them to be more easily detrapped, which enhances their mobility and conductivity [15].
- The secondary electron emission (SEE), which reflects the structure of conduction bands, was highly dependent on the temperature even far below the melting point. The large temperature dependence of the conduction bands in polyethylene is ascribed to the increase in specific volume of the amorphous parts by heating (decrease in the material crystallinity) [43].

The above statement gives more insight into trapping and detrapping of charges in unaged XLPE under electron beam irradiation. Based on this, it is highly suggested that the dominant traps in fresh XLPE samples are deep traps. This is because extensive deep traps perform the almost-trapping charge process, especially at room temperature and at 50 °C. Hence, releasing this charge becomes difficult and sometimes impossible, which explains the nonexistence of discharging currents after the cutoff of the e-beam irradiation.

When it comes to the effect of thermal aging, one can say that physical and chemical changes may be caused by thermal aging on XLPE morphology. The induced chemical and physical alterations significantly affect the material’s localized state density (traps). According to earlier finding of many scholars, long-time aging under temperatures higher than melting temperature of XLPE insulation leads to two main issues affecting the localized state distribution of traps. The first issue is the drastic reduction in the material’s crystallinity [13,31,39]. During aging at 140 °C, the spherulites creating the crystalline parts are completely molten which makes the thermo-oxidative aging easier and destroys progressively, with aging time, these spherulites and the constituted lamellas. Therefore, further harsh crystallinity decrease may be occurred in favor of the amorphous part as the volume of the material is the same. This extending of amorphous region enhances shallow traps generation. The studied material presents a crystallinity decrease from 37.1% to 25.6% and a melting temperature decrease from 108.5 °C to 86.4 °C after 1500 h of aging at 140 °C [31,39].

The second issue concerns the generation of carbonyl groups during aging by oxidation reaction. These carbonyl groups act as shallow traps with energy levels about 0.96 eV [13]. Ouyang et al. have used isothermal surface potential decay (ISPD) tests on the thermally aged XLPE to show that not only new shallow traps are created by thermo-oxidative aging, but there is a conversion of deep traps to shallow ones in XLPE surface. Thus, the existing shallow traps become very shallower.

On the other hand, the polar character of these carbonyl groups enhances both the conductivity of the material [11,37,38] and apparent mobility of charge carriers [11], leading to the leakage current values enhancement after aging.

By increasing the test temperature of thermally aged samples, the detrapping tendency becomes much promoted, and the material will trap less charges. The spreading of charge behavior monitored by the detrapping rate becomes superior to the charge retention one monitored by the trapping rate. Furthermore, increase in temperature leads to a decrease in effective trap energy [28]. The result is the occurrence of detrapping process even when electrons are injected by e-beam irradiation as we have observed in the presented results in Section 4.2.2.

### 5.1. Isothermal Conditions Effect on I_imax_ and I_LS_

**Figure 8:**
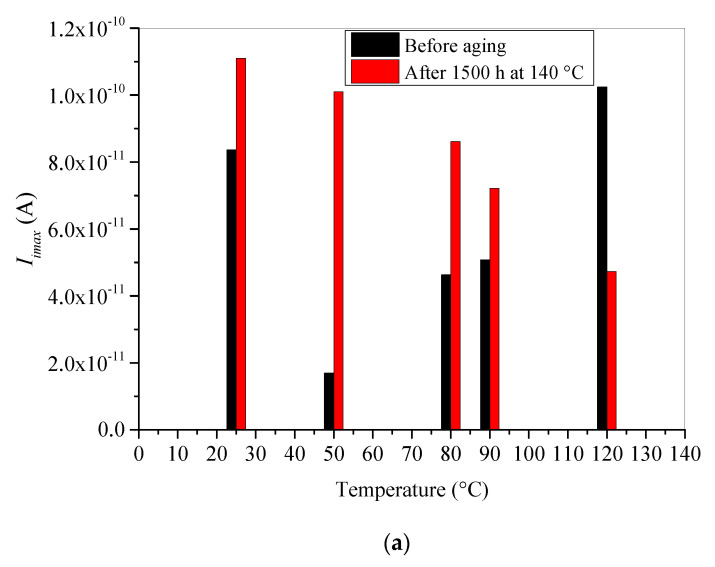

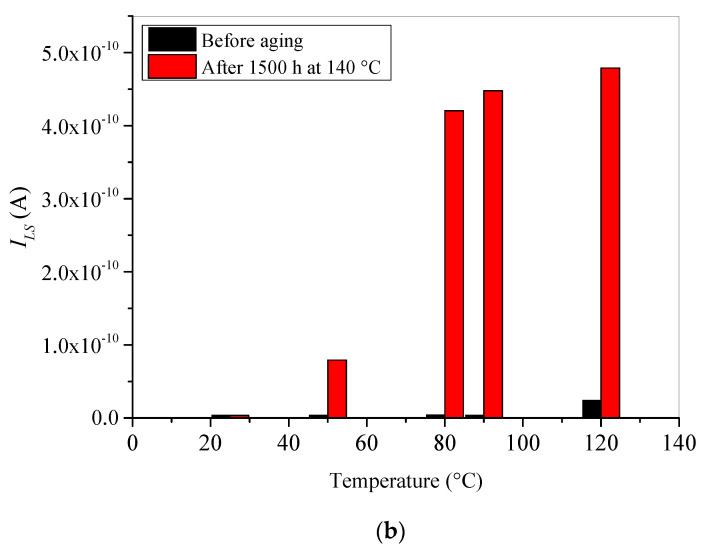
Absolute values of the displacement current at the beginning of irradiation *I_imax_* (**a**) and the leakage current at saturation *I_LS_* (**b**) vs. test temperature.

In order to better investigate the effect of temperature on trapping ability and conduction process of XLPE insulation, the evolution of influence current absolute value at the beginning of irradiation is presented in Figure 8a. *I_imax_* represents the maximum trapping rate. The absolute value *I_LS_* of leakage current at saturation, which represents the conduction process, is presented in Figure 8b. Both electrical parameters (*I_imax_* and *I_LS_*) are presented for unaged and thermally aged XLPE samples in same Figure. From these Figures, one may observe that both characteristics strongly depend on test temperature and thermal aging. 

Figure 8a shows that *I_imax_* for unaged XLPE samples decreases to a minimum at 50 °C and then increases as test temperature increases. Nevertheless, for aged samples it decreases linearly with increasing temperature. The obtained results point out that fresh XLPE material can trap plentifully either at room temperature or at high temperature. At room temperature, the present traps are deep of nature and located at the surface of the material as evidenced by Seggern [41]. Consequently, they can be reached and filled by the injected electrons easily which enhances the trapping rate at the beginning of irradiation.

Additionally, at high temperatures, the material becomes more and more amorphous, which makes the penetration depth of the e-beam into material deeper and the injected electrons may be trapped in shallow traps existing in the amorphous bulk of the material. Thus, a new enhancement of the trapping rate is observed at these high temperatures. Nevertheless, the minimum of *I_imax_* at 50 °C corresponds to minimum of traps density because of the degassing process under vacuum condition leading to elimination of some traps creating by volatile cross-linking by-products. 

Moreover, it was stated here and in other previous papers that thermal aging strongly affects the localized states by creating physical and chemical defects acting as very shallower traps. These traps become more inactive as the applied test temperature increases. Added to this, under high temperatures, the detrapping tendency of the incident electrons increases which makes the trapping ability of the aged XLPE insulation weaker. This statement explains the decrease in maximum trapping rate at the beginning of irradiation with increase in applied test temperature for aged samples. 

The leakage current at saturation *I_Ls_* is depicted in Figure 8b. *I_LS_* increases with increasing applied temperature. This behavior is noticed for both unaged and aged samples. However, the increase in *I_LS_* is more pronounced for the aged sample. It is well known that surface conductivity of dielectrics is activated by temperature, which leads to evacuation to the ground of more free electrons created within the sample surface; hence, *I_LS_* increases with applied temperature increases. The enhanced values of *I_LS_* after aging are due to the presence of carbonyl groups (resulting from thermo-oxidative degradation). With their polar character, these substances enhance material’s conductivity and apparent mobility of charge carriers [37].

### 5.2. Isothermal Conditions Effect on Q_tmax_ and t_c_

**Figure 9:**
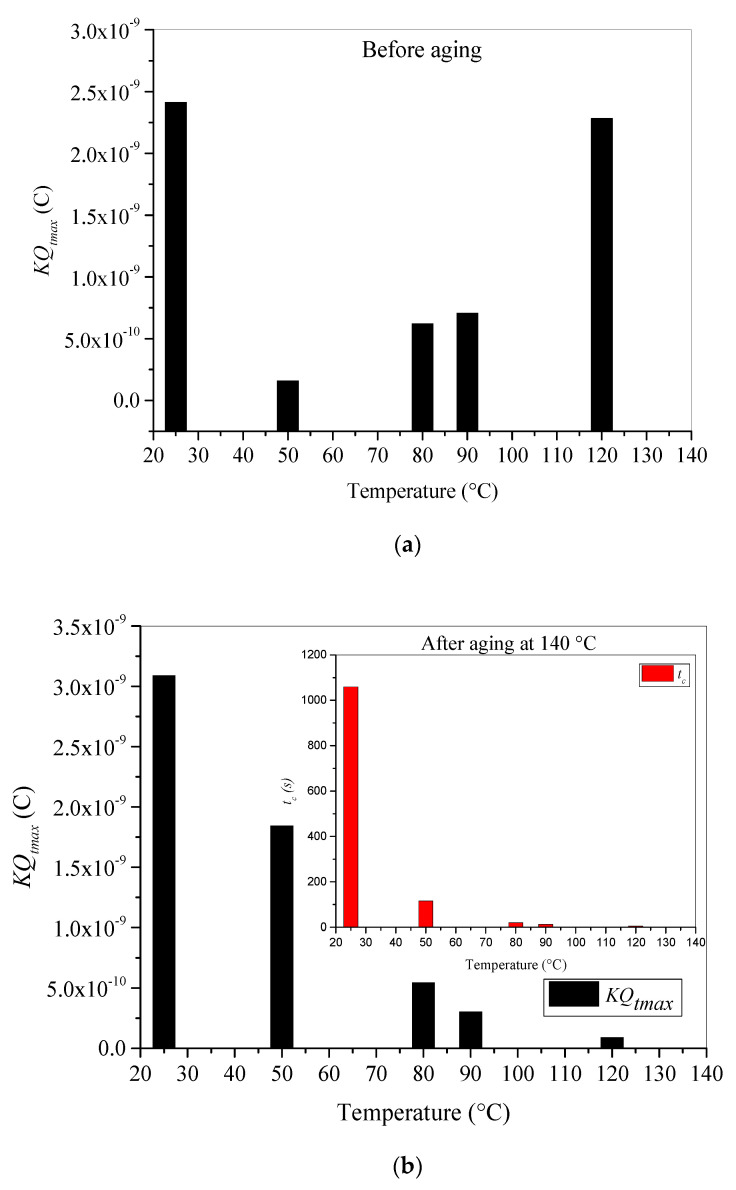
Absolute values of maximum trapped charge during e-beam irradiation step before aging (**a**) and after aging at 140 °C (**b**) vs. test temperature.

The absolute values of maximum trapped charge are presented in Figure 9a,b for unaged and aged samples, respectively. The behavior of this electrical quantity is quite similar to that of *I_imax_*. When the temperature of fresh sample increases from room temperature to 120 °C, the maximum trapped charge decreases to a minimum value at 50 °C and then increases progressively (Figure 9a). However, the maximum trapped charge for aged sample decreases progressively with increasing applied test temperature. In Figure 9b, we present both maximum trapped charge and critical time *t_c_* corresponding to *Q_tmax_*. This critical time decreases also with increasing temperature. 

One can clearly observe in Figure 9a that XLPE material trappes more charges at room temperature and at very high temperatures. The enhanced values of trapped charge at room temperature can be assigned to the extensively existing deep and shallow traps in the XLPE samples after manufacturing [13]. The deep traps generated by the cross-linking by-products resulting from the material’s peroxide cross-linking process. α-methylstyrene, acetophenone, and cumyl alcohol are ascertained by many scholars to be the main volatile cross-linking by-products [35] and create traps having around 1.07 eV as traps depth. Thus, more deep traps in the bulk of the material increases the probability of charge trapping and restrain the migration process of charges. As the experiments are carried out under vacuum conditions (10^−6^ Torr), the increase in temperature from 50 °C to 120 °C leads to a probable degassing process, and the volatile cross-linking by-products may be evaporated progressively, leading to a decrease in deep traps’ density. Hence, the material’s trapping capability is reduced, and the total trapped charge is lower. This assumption is well confirmed in the depicted results in Figure 9a. In addition, another key factor affecting charge trapping process in XLPE insulation is its morphology. Therefore, when the test temperature increases, the amorphous part is extended leading to a decrease in deep traps and increase in shallow traps. According to DSC results [31] and reference [44] the melting process of crystalline parts is started between 60 °C and 70 °C, which well explains the lowest obtained value of trapped charge at 50 °C. At this temperature, the degassing process eliminates part of the deep traps, and the melting process is not started yet, which makes the shallow traps density very small. As temperature rises, the amorphous regions are expended and shallow traps’ density increases, which explains the new increase trends of trapped charge between 80 °C and 120 °C.

For aged material, *Q_tmax_* behavior regarding test temperature becomes different because of the strong changes in the trapping behavior. This latter is related to the nature of existing traps in the material. We have stated that thermal aging at temperatures higher than the melting peak undergoes shallow traps and sometimes very shallow traps. These shallow traps are located on the surface and in the bulk of the material. With increasing temperature, the effective energy of shallow traps is reduced, making them inactive; hence, the trapped electrons can be easier detrapped with thermal energy. Furthermore, as the temperature increases, the release of trapped electrons becomes faster, reducing the level of charging.

In consequence, the maximum trapped charge decreases with increasing applied temperature. Furthermore, the enhancement of both material conductivity and apparent mobility of the charge carriers caused by thermal aging leads to a weaker trapping rate of charges and a greater evacuating rate of electrons to the ground. As a result, a reduction in maximum trapped charge (Figure 9b) and enhancement of leakage current at steady state (Figure 8b) are noticed. 

### 5.3. Isothermal Conditions Effect on Electrostatic Influence Factor K

**Figure 10:**
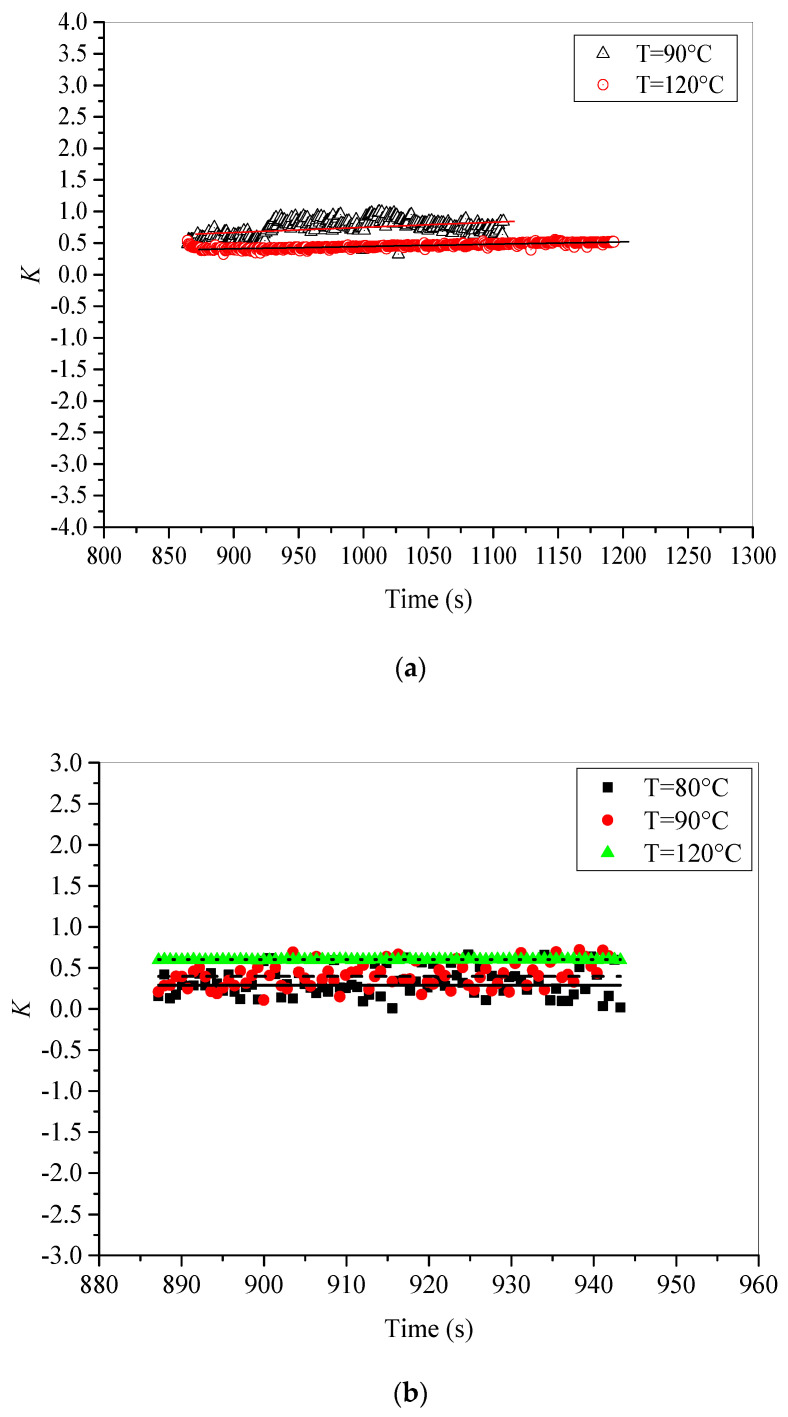
Evolution of electrostatic influence factor *K* vs. test temperature, (**a**) before aging, and (**b**) after aging at 140 °C.

The electrostatic influence factor *K* is calculated by Equation (4) during the discharging step using time evolution of both influence and leakage currents. The obtained results for unaged and thermally aged samples are depicted in Figure 10a,b, respectively. The calculation leads us to obtain acceptable results for only high temperatures. The mean value of *K* remains constant for each temperature. This behavior is observed in both unaged and aged samples. It seems that thermal aging did not have a considerable effect on the factor *K*.

The factor *K* decreases with increasing temperature for unaged samples. However, a reverse behavior is observed in the case of aged samples, where the increase in temperature leads to a rise in the factor *K*. The obtained results suggest that behavior of *K* versus applied temperature is strongly related to the nature and the positions of traps. If we take the fresh sample, the previous analysis stated that deep traps govern the behavior of trapping and detrapping of charges remains valid here. Since deep traps are located in the surface of the material, and the increase in temperature may reduce the effective depth of these traps, the volume of interaction spreads out and gets closer to the surface, leading to a reduction in the factor *K*. However, if we consider the aged sample, shallow traps are dominant and located deeper in the material, which makes the interaction process denser as the temperature increases leading to enhancement of the factor *K*. 

### 5.4. Isothermal Conditions Effect on Total Electron Emission Yield σ

Since secondary electron emission (SEE) is one of the mechanisms regulating charge’s trapping and detrapping processes subjected to e-beam irradiation, its sensitivity on the applied temperature and XLPE thermal aging is studied in this section. We recall here that total electron emission yield *σ* is the ratio between the total number of electrons emitted by the surface and the number of impinging electrons. Mathematically, it can be deduced from the given Equation (1) in Section 2.1 [29]:

(7) $$\sigma \left(t\right)=\frac{I_{\sigma }\left(t\right)}{I_{0}}=1-\frac{I_{L}\left(t\right)}{I_{0}}-\frac{I_{i}\left(t\right)}{kI_{0}}$$

**Figure 11:**
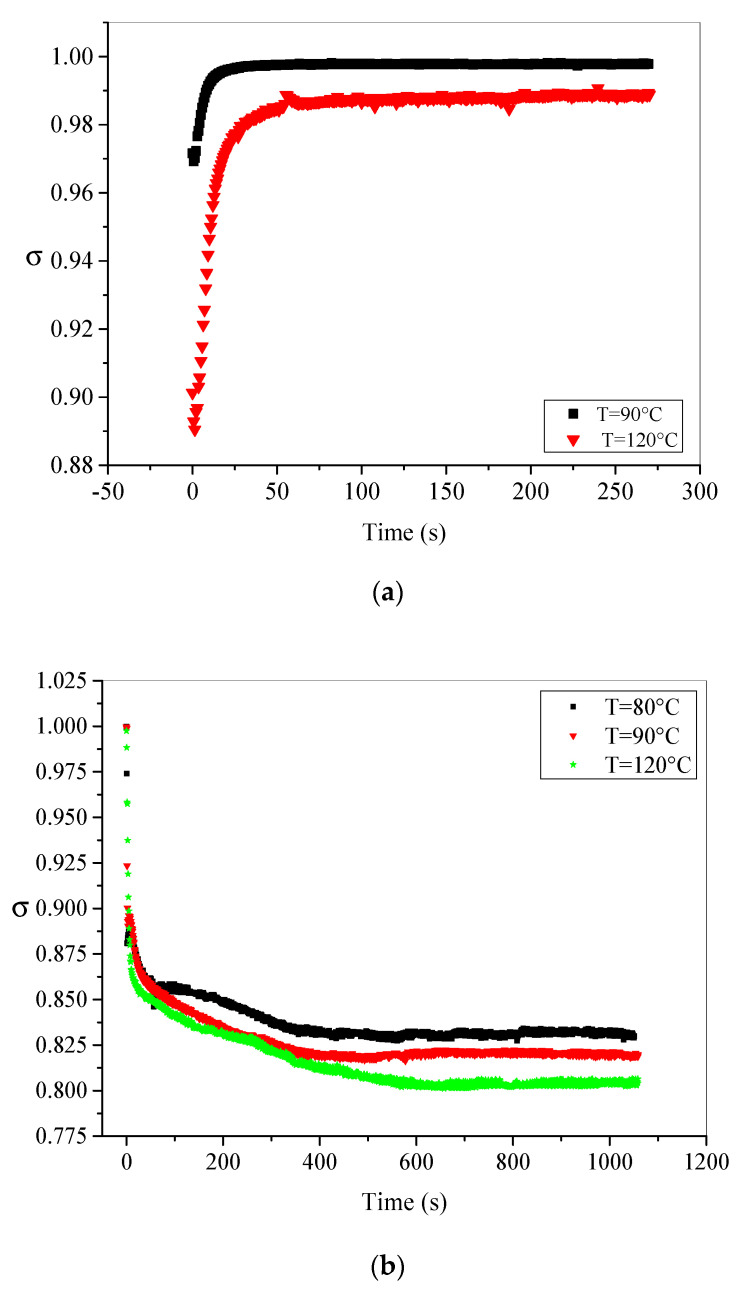
Evolution of total secondary electron emission yield *σ* vs. test temperature, (**a**) before aging, and (**b**) after aging at 140 °C.

The time evolutions of *σ* for unaged and aged samples at different temperatures are plotted in Figure 11a,b, respectively.

From Equation (7), it is clear that the computation of *σ* requires knowledge of the *K* value; hence, the values of *σ* are also computed only at high temperatures. In the case of fresh material (Figure 11a), *σ* presents an increasing behavior from an initial (minimal) value (*σ*_0_) at beginning of irradiation to reach a steady state having stationary value *σ_s_*. This behavior is observed for all applied test temperatures and is mainly assigned to slowing down of incident electrons caused by the external electrical field induced by the negative charging (trapped charge is negative) [29] and the secondary emission yield curve given in [22,45]. Theoretically, total electron emission yield may evolve towards the unity if the leakage current is negligible when the dynamic equilibrium is reached (we assume that influence current is equal to zero at the equilibrium). However, this situation is not satisfied for the unaged XLPE material and *σ* does not reach the unity because leakage current has some contribution in the regulation mechanism of charge trapping process. Thus, the stationary value *σ_s_* is given as follows:

(8) $$\sigma_{s}=1-\frac{I_{Ls}}{I_{0}}$$

Furthermore, it can be seen also that *σ_s_* decreases with the temperature increases. This tendency fits well with other semi-crystalline polymers used in previous studies [28,29]. Generally, secondary electron emission depends on the sample’s specific crystalline state, particularly on the interaction of generated SEE with electrons, phonons, or crystalline defects [26]. By increasing temperatures near to melting temperature of XLPE material, the amorphous part is expended, and more defects are created by the disorder’s enhancement in the material. Consequently, the interactions during secondary electrons transport towards the surface are enhanced, leading to a reduction in *σ_s_*. The computed values for different temperatures are listed in Table 1.

Besides, the depicted results in Figure 11b show reverse behavior of *σ* for thermally aged material at 140 °C. In this case, *σ* decreases with irradiation time from maximum at the beginning of irradiation (*σ*_0_) toward stationary state (*σ_s_*). This behavior is observed for all applied test temperatures. The decreasing trend of *σ* for aged sample can be explained by sample’s positive charging caused by the detrapping process prevailing, even under e-beam irradiation, at high temperatures.

This statement is supported by the interesting finding in Cazaux’s work [45,46,47] on the secondary electron emission making certain that negative charging leads to *σ* increase (as in the case of unaged sample) and positive charging leads to *σ* decrease (as in the case of aged sample). At early beginning of irradiation, the contribution of *I_L_* and *I_i_* in Equation (7) is very limited because of their very low values, so *σ* takes its maximum value (*σ*_0_) practically equal the unity as we can see in Figure 11b. However, as the irradiation progresses with time, leakage current becomes very important (see Figure 4a) and its contribution monitors *σ* evolution. This suggestion is supported by the very similar evolution (in shape) of leakage current (Figure 4a) and *σ* (Figure 11b). The computed values of *σ_s_* are also listed in Table 1 and show the reduction in this yield with increase in temperature. It is very clear from the listed values in Table 1 that *σ_s_* values of aged material are lesser than those of unaged material. This is highly probable due to many factors such as: (i) enhancement of defects in the crystalline structure, (ii) enhancement of carbonyl groups caused by thermo-oxidative degradation in the material surface, (iii) morphological defects (creation of craters and holes in the surface of the sample that can have different shapes and sizes). All these defects interact successfully with secondary electrons which allow them to lose big part of their kinetic energy. Finally, they hardly reach the surface and cannot be evacuated outside of the material. The amount of electrons reaching the surface are transported by the extensive created charge carriers under thermal aging to the ground, hence the enhancement of the leakage current and the reduction in *σ_s_*.

## 6. Conclusions

This study has reported the effect of isothermal conditions and thermal aging on trapping and detrapping processes of e-beam irradiated XLPE insulation in SEM. These processes are monitored by influence and leakage currents, and other derived amounts such as trapped charge. The obtained sound finding leads to ascertain many significant conclusions. 

Firstly, since the trapping and detrapping processes are closely related to the morphology in XLPE material, the temperature and thermal aging significantly affect this morphology by altering the distribution of aggregation in existing deep and shallow traps. Furthermore, it has been pointed out that deep traps govern the process in the unaged material, whereas shallow traps take part in the aged material. 

For the unaged material, the increase in temperature leads to the following: (i) A decrease and then an increase in maximum trapped charge (*Q_tmax_*) and maximum trapping rate at the beginning of irradiation (*I_imax_*). A minimum has been noticed at 50 °C and corresponds to the reduction in traps density caused by a highly probable elimination, with a degassing process of some volatile cross-linking byproducts acting as deep traps. (ii) Not any released charges during the discharging step at room temperature; nevertheless, part of the trapped charge has been lost at high temperature under the effect of thermal energy. (iii) A moderate increase in leakage current at steady state (*I_LS_*) especially at 120 °C because of the thermal activation of the surface conductivity. (iv) A reduction in electrostatic influence factor (*K*) and total electron emission yield at steady state (*σ_s_*).

For aged material at 140 °C, the increase in temperature causes the following: (i) A decrease in maximum trapped charge (*Q_tmax_*) and maximum trapping rate at early stage of irradiation (*I_imax_*). This behavior is caused by the enhancement of shallow traps density after thermal aging. The new brought structure of XLPE material traps fewer charges as the temperature rises. A detrapping process has been observed even during the charging process, except at room temperature, where part of the trapped charge has been released. The obvious detrapping process is pronounced during the discharging process. (ii) A sharp increase in leakage current (*I_LS_*), especially at high temperature (more than 80 °C), because of the enhancement of charge carriers created by the aging process. (iii) An increase in electrostatic influence factor (*K*) and a decrease in total electron emission yield at steady state (*σ_s_*).

## Figures and Tables

**Table 1:** Computed values of *σ*_0_ and *σ_s_* for different cases.

Temperature (°C)	Unaged Material		Aged Material at 140 °C	
	*σ*_0_	*σ_s_*	*σ*_0_	*σ_s_*
80 °C	-	-	~1	0.8322
90 °C	0.9693	0.9979	~1	0.8192
120 °C	0.9012	0.9889	~1	0.8060

## Data Availability

Not applicable.
